# Blood glucose and lactate levels as early predictive markers in patients presenting with cardiogenic shock: A retrospective cohort study

**DOI:** 10.1371/journal.pone.0306107

**Published:** 2024-07-25

**Authors:** Hannah Billig, Muntadher Al Zaidi, Florian Quacken, Jan Görtzen-Patin, Philip Roger Goody, Ingo Gräff, Georg Nickenig, Sebastian Zimmer, Adem Aksoy

**Affiliations:** 1 Department of Cardiology—University Hospital Bonn, Bonn, Germany; 2 Hausarztzentrum Rheinbach, Rheinbach, Germany; 3 Department of clinical acute- and emergency medicine, University Hospital Bonn, Bonn, Germany; Albert Einstein College of Medicine, UNITED STATES

## Abstract

Lactate and glucose are widely used biochemical parameters in current predictive risk scores for cardiogenic shock. Data regarding the relationship between lactate and glucose levels in cardiogenic shock are limited. Thus, we aimed to analyze glucose and lactate as early markers for in-hospital mortality in cardiogenic shock. In this retrospective cohort study, 312 patients presenting with cardiogenic shock to a tertiary-care hospital between 2016 and 2018 were included. Apparent cardiogenic shock was defined as hypoperfusion with hemodynamic compromise and biochemical marker increase due to diminished tissue perfusion, corresponding to SCAI shock stages. In-hospital mortality was assessed as the primary endpoint. The median age of the study population was 71 (60–79) years and the etiology of cardiogenic shock was acute myocardial infarction in 45.8%. Overall in-hospital mortality was 67.6%. In the receiver operating curve analysis, the area under the receiver-operating curve (AUC) for prediction of in-hospital mortality was higher for lactate (AUC: 0.757) than for glucose (AUC: 0.652). Both values were significantly associated with outcome (groups created with best cutoff values obtained from the Youden index). Correlation analysis showed a significant non-linear association of both values. In a multivariable stepwise Cox regression analysis, lactate remained an independent predictor for in-hospital mortality, whilst glucose, despite being implicated in energy metabolism, was not independently predictive for mortality. Together, these data suggest that lactate at admission is superior for mortality prediction in patients with apparent cardiogenic shock. Glucose was not independently predictive for mortality.

## Introduction

Cardiogenic Shock (CS) is a critical syndrome of systemic hypoperfusion and tissue hypoxia associated with high mortality [1]. In-hospital mortality rates of 40–62% have been reported [2], and therefore, early identification of the underlying cause of CS is crucial. In acute myocardial infarction (AMI) complicated by CS, early revascularization of the culprit lesion can improve survival [3, 4]. As for pharmacological support, vasopressors are frequently needed to maintain tissue perfusion in hemodynamically unstable patients [5]. Despite a lack in high-quality evidence, mechanical circulatory support has been increasingly implemented to improve cardiac output and support end-organ perfusion. High complication rates demand careful patient selection and multidisciplinary expertise [6].

Early assessment of outcome is needed for therapeutic decision making. Several scores for prediction of early mortality in CS patients have been introduced [7–10]. Within these scores, glucose and lactate have been increasingly recognized as valuable predictive markers in CS. Elevated lactate levels are a hallmark of poor tissue perfusion and metabolic distress, and have been shown to be associated with increased morbidity and mortality in patients with cardiac disease.

In a physiological state, glucose is the primary source of energy for most cells and is generally metabolized through aerobic glycolysis and the citric acid cycle [11, 12].

Contrary, in hypoxic phases (e.g. tissue hypoperfusion during CS), glucose can also be metabolized in the absence of oxygen. This anaerobic glycolysis is less efficient, however, it allows cells to generate energy quickly in the absence of oxygen [11]. In CS, hyperlactatemia is most likely caused by an increase in lactate production, instead of an impaired lactate clearance [13, 14].

In recent years, several studies have demonstrated the utility of glucose and lactate as predictive markers in CS, and especially lactate is widely used to guide the management and estimate prognosis of these critically ill patients. Lactate as well as its clearance in a time span of 6–8 hours have been shown to be valuable prognostic markers to predict early prognosis in CS [9, 15–18]. The role of lactate has been extensively studied across diverse patient populations with varying etiologies, including those on mechanical support [17, 19, 20] and those who have experienced cardiac arrest [21–23].

Notably, the majority of research on this topic has been confined to patients experiencing AMI complicated by CS or have excluded patients after cardiac arrest [24–29]. However, a limited number of studies analyzed glucose in a real-world-setting of CS, encompassing various etiologies and severity levels, from beginning CS characterized by hypotension and signs of organ hypoperfusion to severe cases requiring resuscitation [30, 31].

Data comparing usefulness and relationship of both lactate and glucose are limited. The study of Kataja et al. examines the role of admission blood glucose levels as a predictive marker for in-hospital mortality in patients with CS due to AMI or other etiologies and with or without cardiac arrest. The study does mention correlations between glucose levels and other clinical markers, including lactate. Severe hypo- and hyperglycemia is associated with higher lactate levels. However, it does not perform a direct comparative analysis of their usefulness or a detailed exploration of their interrelationship beyond correlational observations [30].

We hypothesized that glucose could serve as an earlier or a complementary marker to lactate for in-hospital mortality in CS.

## Methods

### Study design and population

We conducted a retrospective, single-center analysis at our tertiary academic center. Patients with cardiogenic shock treated from 2016 to 2018 were included. For this purpose, all patients with ICD-10 code R57.0 were screened for the presence of classic cardiogenic shock.

Inclusion criteria were hemodynamic relevance (defined as hypotension < 90 mmHg or support by catecholamines) and biochemical detectability (defined as lactatemia > 2 mmol/l) of shock. By that, we identified patients exhibiting clinical signs of cardiogenic shock, which correspond to stages B through E of the SCAI shock classification.

Exclusion criteria were age younger than 18 years or treatment in a different department because of the variety of documentation systems at our site.

Because glucose and lactate were aimed to be studied as predictors of short-term outcome, in-hospital mortality was chosen as the primary end point. Patients without baseline glucose and lactate measurements were excluded.

This retrospective study involving human participants was in accordance with the ethical standards of the institutional and national research committee and with the 1964 Helsinki Declaration and its later amendments or comparable ethical standards. The Human Investigation Committee (IRB) of University of Bonn confirmed that consultation of the committee is not required for our retrospective evaluation of data obtained in the course of routine diagnostics / care (Institutional Review Board approval number: 243/22, approval date: June 14, 2022). As data were analyzed retrospectively and data were analyzed anonymously, no consent has been obtained from the patients.

### Data collection

Demographic and clinical data, past medical history, and laboratory values were obtained from the electronic medical record system from July-November 2022. F.Q., H.B. and A.A. had access to individual participant information during data collection. After data collection, information was anonymized.

### Statistical analysis

SPSS software version 29 and R 4.4.0. were used for the statistical analyzes.

Because most metric variables were not normally distributed, statistics for all metric variables are shown as median with interquartile range (IQR). Baseline characteristics were tested using the Mann-Whitney U test for metric variables and the chi-square test for categorical variables.

Receiver-operating curves (ROCs) with the corresponding area under the curve (AUC) were calculated for the first glucose and lactate value after hospital admission. The best cut-off values for predicting in-hospital mortality were determined using the Youden index. An association of the groups with the primary end point was examined by Kaplan-Meier-curves and the log-rank test.

Relationship of lactate and glucose levels were tested with the Spearman’s rank correlation coefficient. Strength, direction and form of the correlation was visualized using scatter plots.

Cox regression analysis was used to identify predictors of the primary end point. For this, baseline variables showing an association with in-hospital mortality (age, prior myocardial infarction, arterial hypertension, haemoglobin, platelets, pH, glucose, and lactate) were included using a stepwise-forward entry approach with an entry level of <0.05.

### Power analysis

A post-hoc power analysis was conducted to determine the study’s ability to detect differences in lactate and glucose levels between survivors and non-survivors of cardiogenic shock. The analysis utilized a sample size of 312 patients, with effect sizes of Cohen’s d = 1.015 for lactate and d = 0.532 for glucose. The significance level was set at 0.05 with a two-sided hypothesis test. The results indicated that the study was sufficiently powered, achieving near-complete power (100% for lactate and 99.99% for glucose) to reliably detect significant differences, ensuring the robustness of the findings.

## Results

Demographic and clinical characteristics of study participants (overall, and stratified by survival) are summarized in Table 1. The analysis of baseline characteristics was exploratory, intended to identify potential patterns or differences that may warrant further investigation. Overall, the median age of patients was 71 (60–79) years and 76.6% were male. Within the cohort, there was a high prevalence of cardiovascular risk factors (33.3% with diabetes, 63.2% with arterial hypertension, 34.6% were active or former smokers) and ischemic heart disease (27.6%).

**Table 1:** Baseline characteristics.

	Overall (n: 312)	Survivors (n: 101)	Non-survivors (n: 211)	p-value^a^
Sex (male), n (%)	239 (76.6)	82 (81.2)	157 (74.4)	0.186
Age	71 (60–79)	65 (55–76)	73 (61–80)	**< 0.001**
Left ventricular ejection fraction [%]	40 (25–40)	35 (20–47)	40 (30–53)	0.072
Cardiac Arrest at presentation, n (%)	207 (66.6)	54 (54)	153 (27.5)	**0.001**
Bystander resuscitation, n (%)	78 (31.8)	29 (35.8)	49 (29.9)	0.349
On mechanical ventilation, n (%)	227 (76.9)	70 (72.2)	157 (79.3)	0.749
Mechanical circulatory support				
Use of any MCS, n (%)	67 (21.5)	22 (21.8)	45 (21.3)	0.927
• Impella, n (%)	47 (15.1)	18 (17.8)	29 (13.7)	
• IABP, n (%)	1 (0.3)	0	1 (0.5)	
• VA-ECMO, n (%)	14 (4.5)	3 (3)	11 (5.2)	
• ECMELLA, n (%)	5 (1.6)	1 (1)	4 (1.9)	
Etiology of CS				
AMI, n (%)	143 (45.8)	58 (57.4)	85 (40.3)	**0.004**
Non-AMI	169 (54.2)	43 (42.6)	126 (59.7)	
• Aortic valve stenosis, n (%)	10 (3.2)	1 (1)	9 (4.3)	
• Decompensated chronic heart failure, n (%)	14 (4.5)	6 (5.9)	8 (3.8)	
• Tachycardia/bradycardia, n (%)	34 (10.9)	16 (15.8)	18 (8.5)	
• Cardiomyopathy, n (%)	14 (4.5)	10 (9.9)	4 (1.9)	
• Pulmonary artery embolism, n (%)	13 (4.2)	5 (5)	8 (3.8)	
• Myocarditis, n (%)	2 (0.6)	1 (1)	1 (0.5)	
Medical history				
Ischemic heart disease, n (%)	86 (27.6)	78 (77.2)	63 (29.9)	0.19
Heart Failure, n (%)	50 (20.7)	18 (18.2)	32 (22.4)	0.428
Diabetes, n (%)	81 (33.3)	25 (25.5)	56 (38.6)	**0.033**
Arterial hypertension, n (%)	158 (63.2)	58 (58.6)	51 (33.8)	0.221
Ever-Smoker, n (%)	83 (34.6)	34 (35.1)	49 (34.3)	0.9
Laboratory findings				
GFR [ml/min]	65 (36–70)	69 (43–70)	57 (35–70)	0.235
ALT [U/l]	78 (38–188)	73 (36–191)	78 (38–188)	0.733
White blood cell count [G/l]	12.79 (9.68–17.2)	13.25 (10.18–17.2)	12.68 (9.46–17.23)	0.583
Haemoglobin [g/dl]	12.1 (10.3–13.9)	12.9 (11.5–14.8)	11.6 (9.8–13.2)	**< 0.001**
pH	7.222 (6.97–7.373)	7.343 (7.212–7.402)	7.134 (6.875–7.343)	**< 0.001**
Glucose [mg/dl]	231 (154–334)	191 (135–268)	262 (167–367)	**< 0.001**
Lactate [mmol/l]	7.12 (2.95–11.72)	3.41 (2.01–6.9)	9.25 (4.51–12.89)	**< 0.001**
Vital signs after ICU admission				
Mean arterial pressure [mmHg]	76 (66–90)	82 (71–90)	73 (62–90)	**0.026**
Heart rate [BPM]	82 (71–108)	80 (68–94)	88 (75–111)	**0.014**

AMI acute myocardial infarction

^a^Bonferroni correction was used to correct for multiple testing.

The primary endpoint (in-hospital mortality) occurred in 211 (67.6%) patients. In 143 (45.8%) patients, the etiology of cardiogenic shock was acute myocardial infarction (AMI). For patients with AMI, survival rates were significantly higher (57.4%) than in non-AMI-etiologies (40.3%). 207 (66.6%) patients experienced initial cardiac arrest (during prehospital care or in the emergency department). Mechanical circulatory support (VA-ECMO, Impella or intra-aortic balloon pump) was applied in 67 (21.5%) patients.

Mean left ventricular ejection fraction (LVEF) was 40% (25–40%) and did not differ significantly between survivors and non-survivors.

Initial mean arterial pressure was higher in survivors than in non-survivors (82 [71–90] vs. 73 [62–90] mmHg, p 0.026) whilst heart rate was lower (80 [68–94] vs. 88 [75–111] BPM, p 0.014; Table 1).

**Fig 1:**
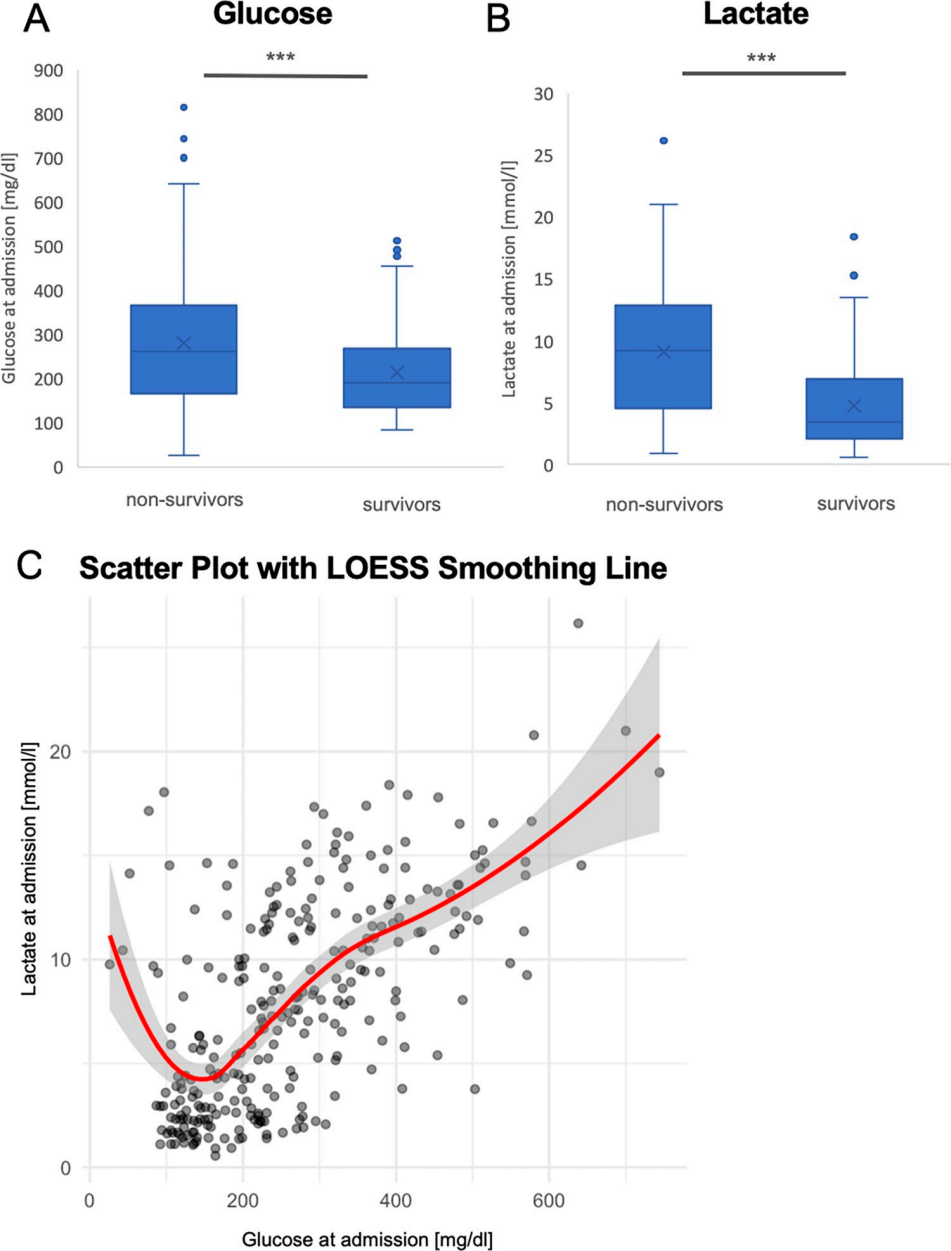
Boxplots and scatter plot for lactate and glucose levels, displayed for survivors or non-survivors. A) Boxplot for glucose at admission, stratified for in hospital mortality. B) Boxplot for lactate at admission, stratified for in hospital mortality. C) scatter plot with LOESS smoothing line for glucose and lactate values at admission.

Baseline values for haemoglobin (Hb) and pH were significantly lower for non-survivors than for survivors (Hb: 11.6 [IQR 9.8–13.2] vs. 12.9 [IQR 11.5–14.8] g/dl, p < 0.001; pH: 7.134 [IQR 6.875–7.343] vs. 7.343 [7.212–7.402], p < 0.001; Table 1). Glucose and lactate differed significantly between non-survivors and survivors (glucose: 262 [IQR 135–367] vs. 191 [IQR 135–268] mg/dl, p < 0.001, lactate: 9.25 [IQR 4.51–12.89] vs. 3.41 [IQR 2.01–6.9] mmol/l, p < 0.001; Table 1, Fig 1A and 1B).

To test the relationship between glucose and lactate values in survivors and non-survivors, we performed correlation analyses. The results indicated a significant non-linear relationship between lactate and glucose values, as visualized by a scatter plot with a LOESS smoothing line (Fig 1C). This non-linear relationship was confirmed by a Generalized Additive Model, which demonstrated that the smooth term for glucose was highly significant (p < 0.001), indicating a complex, non-linear interaction between these biomarkers.

### Univariate analysis of in-hospital mortality

**Fig 2:**
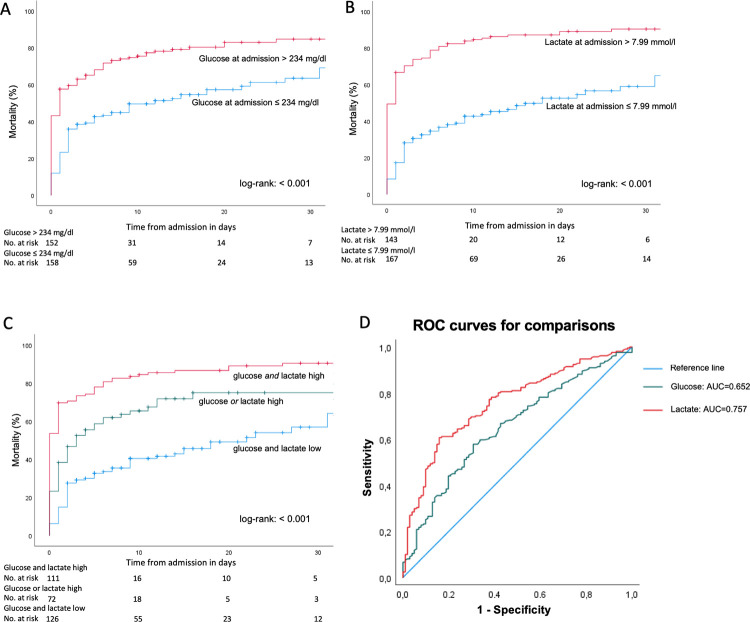
Survival analysis. Kaplan-Meier curves for time from admission to death stratified for calculated best cut-off values. A) for glucose at admission. B) for lactate at admission. C) Kaplan-Meier curves for time to death stratified for a score of consisting of glucose and lactate best cutoff values. D) ROC Curves in Prediction of In-Hospital-Mortality for baseline lactate and baseline glucose.

To compare mortality prediction by baseline glucose and lactate values, receiver operating curves (ROC) were generated (Fig 2D). The area under the ROC for glucose was 0.652 (95% CI 0.588–0.715), while it was 0.757 for lactate (95% CI 0.702–0.812). The DeLong test revealed a significant difference between these AUCs (p < 0.001, 95% CI for the difference in AUC: -0.161 to -0.049).

The Youden index was used to obtain best cutoff values for predicting in-hospital mortality. For glucose, the optimal cutoff value was 234 mg/dl (sensitivity 0.573, specificity 0.693) while it was 7.99 mmol/l for lactate (sensitivity 0.607; specificity 0.842).

When comparing in-hospital mortality according to the obtained cutoff values, both glucose and lactate were found to be significant predictors for in-hospital mortality (Kaplan-Meier curves, Fig 2A and 2B).

To test if a combination of glucose and lactate values can further improve mortality prediction, we created a score out of both parameters: glucose > 234 mg/dl (high) and lactate > 7.99 mmol/l (high) = 2 points; glucose or lactate high = 1 point; both glucose and lactate < 234 mg/dl or 7.99 mmol/l respectively = 0 points. The score was highly significant in predicting in-hospital mortality for the 3 groups, but it did not outperform outcome prediction obtained by baseline lactate alone (Fig 2C).

**Fig 3:**
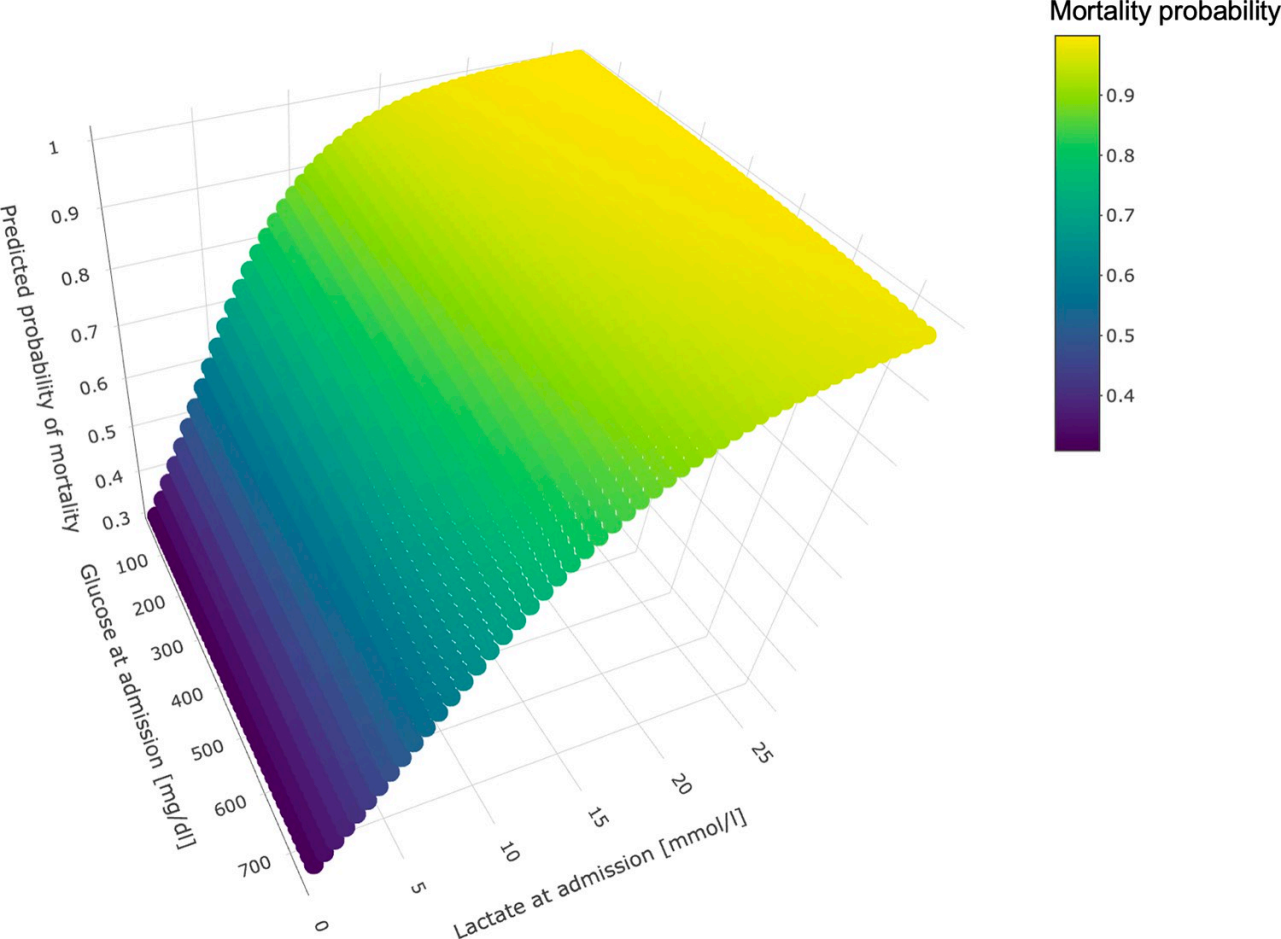
3-Dimensional prediction plot. Prediction plot as a function of lactate (x-axis) and glucose (y-axis), with the mortality probability on the z-axis.

A 3-dimensional (3D) plot illustrates the relationship between glucose and lactate levels at admission, and the predicted probability of mortality for patients (Fig 3). The 3D plot suggests that while high glucose levels alone (without elevated lactate) do not significantly increase mortality risk, high lactate levels alone (even without high glucose) do increase mortality risk. This indicates the dominant role of lactate as a prognostic marker in predicting mortality among the patient our study population.

We also performed decision curve analysis to evaluate the clinical usefulness of lactate and glucose cut-offs in predicting survival outcomes. The results indicated that the lactate cut-off provided a greater net benefit across a range of threshold probabilities compared to glucose. These findings are detailed in the supplementary material (S1 Fig).

### Cox regression analysis

In a multivariable, stepwise Cox regression analysis, age (HR 1.039; 95% CI 1.018–1.06) and lactate (HR 1.829; 95% CI 1.005–3.33) were independently associated with the primary endpoint (Table 2). In the Cox regression analysis, glucose was not significantly predictive for in-hospital mortality (HR 1.19; 95% CI 0.679–2.083; p-value 0.544). Cardiac arrest at presentation, CS due to myocardial infarction, diabetes, baseline hemoglobin and pH were not significantly predictive in the Cox regression analysis.

**Table 2:** Multivariate, stepwise Cox-regression analysis.

	Hazard Ratio	95% CI	p-value
**Male Sex**	0.821	0.489–1.378	0.455
**Age per year**	1.039	1.018–1.06	**< 0.001**
**Cardiac Arrest at presentation**	0.717	0.42–1.225	0.224
**Myocardial infarction**	1.137	0.731–1.767	0.569
**Diabetes**	1.269	0.793–2.029	0.321
**Heart rate**	1.006	0.996–1.0016	0.216
**Mean arterial pressure**	1.003	0.99–1.017	0.658
**Baseline haemoglobin > 12.2 g/dl**	0.822	0.51–1.323	0.419
**Baseline pH > 7.281**	0.6	0.343–1.05	0.073
**Baseline glucose > 234 mg/dl**	1.19	0.679–2.083	0.544
**Baseline lactate > 7.99 mmol/l**	1.829	1.005–3.33	**0.048**

CI conficende interval

To further examine a possible non-linear relationship between glucose and lactate with survival outcomes, we performed a Restricted Cubic Splines (RCS) model. The RCS model confirmed the significant impact of lactate on survival outcomes, indicating a primarily linear relationship (non-linear p = 0.579). Conversely, glucose levels did not exhibit significant non-linear effects and maintained a largely linear or non-significant relationship with survival outcomes (p-value = 0.5469; non-linear p = 0.443). Detailed results of the RCS analysis are provided in the supplementary data (S3 Table).

## Discussion

The major findings of this retrospective study are: first, there is a significant non-linear relationship between glucose and lactate at baseline in patients with cardiogenic shock (CS). Second, despite this pronounced association, only lactate was an independent predictor for in-hospital mortality.

Glucose and lactate have increasingly been recognized as predictive markers for mortality in cardiogenic shock as well as other cardiac and non-cardiac diseases [7, 9, 27, 30, 32, 33]. Two studies investigated the origin of hyperlactatemia in shock: Levraut *et al*. suggested that in patients with septic shock that have already been weaned from catecholamines, hyperlactatemia resulted from a reduction of lactate clearance [34]. Revelly *et al*. investigated hyperlactatemia in patients with septic or cardiogenic shock and compared the results to a cohort of healthy subjects. In contrast to Levraut *et al*., they found that hyperlactatemia was mainly caused by an increase of lactate production, while lactate clearance was not substantially different in shock patients [7]. Beside possible methodological differences between the two studies, clinical discrepancies between both cohorts may explain the differing results. First, lactate metabolism may be essentially different in shock situations from different causes. Second, the application of catecholamines, such as epinephrine, may contribute substantially to lactate production through β2-receptor stimulation and consecutive cAMP-production [14].

In acute illness, hyperglycemia is caused by extensive gluconeogenesis due to hypothalamic-pituitary-adrenal and sympatho-adrenal response [35]. Although the elevation of glucose levels is a part of the body’s physiologic response to stress, extensive hyperglycemia at admission is implicated with a worse prognosis and it has been associated with worse outcomes across various clinical scenarios, including heightened inflammation and subsequent complications, impaired left-ventricular function and greater myocardial damage in patients AMI [36, 37].

Under the assumption that hyperlactatemia is secondary to hyperglycaemia in CS, we hypothesized that glucose might be an earlier or a synergistic marker for outcomes in CS. However, our results showed that only lactate was a significant prognostic parameter in patients with CS, while glucose did not show significant predictive value in the Cox regression model. Correlation analyses revealed a complex non-linear relationship of both parameters.

The predictive value of glucose may differ between patients with and without previously recognized diabetes. Analysis of 141 680 patients with AMI showed that only in patients without diabetes, elevated glucose values were associated with a linear increase of mortality [27]. These findings have been confirmed by Yang et al. who investigated the prognostic value of blood glucose levels in 816 patients with and without diabetes and with CS as a consequence of AMI [26]. Glucose was predictive for 30-day mortality only in patients without known diabetes. The CardShock trial investigated glucose levels in 212 patients presenting with apparent CS of different etiologies from 2010–2012. The cohort experienced cardiac arrest in only 28% and the etiology was acute coronary syndrome in 81%. After separation of the cohort to five distinct groups of hyperglycaemia, glucose showed a negative predictive value for the group with the most severe hyperglycaemia. However, the authors found that diabetes modified the prognostic value of admission blood glucose [30].

For patients with AMI, survival rates in our cohort were significantly higher (57.4%) than in those with non-AMI etiologies (40.3%). The observation that AMI was more prevalent among survivors is intriguing and may be attributed to several key factors. Firstly, patients with AMI often present with more recognizable symptoms, leading to rapid diagnosis and treatment, including timely interventions such as revascularization, which significantly improve survival outcomes. Secondly, the clinical pathways for managing AMI are well-established, offering a clear protocol for immediate treatment. In contrast, other etiologies of cardiogenic shock may lack such well-defined treatment protocols, potentially delaying critical interventions and contributing to lower survival rates.

Patients with impaired glucose metabolism or diabetes are known to have a higher risk for mortality when presenting with AMI alone or AMI complicated by CS [38–40]. As diabetes has a high prevalence in patients with CS, identification of prognostic parameters is crucial for risk evaluation. In contrast to recent studies, we studied a cohort of patients with cardiogenic shock of various etiologies. In line with the results for patients with AMI, we could demonstrate that glucose values are not significantly predictive for short-term-outcomes in our cohort.

Medical history or the origin of CS is infrequently known in patients who are referred to the emergency department. In this acute setting, comprehensively applicable prognostic parameters are of great relevance.

In comparison to most other investigations, our cohort comprised a more severe state of CS with a majority (66.6%) presenting with cardiac arrest and mechanical circulatory support being applied in 21.5%. Only 45.8% experienced CS because of AMI. In this critically-ill cohort, lactate values at admission showed better discriminative characteristics for in-hospital mortality than glucose (AUC, Kaplan-Meier curves). The combination of both, baseline glucose and lactate in a score did not add predictive value in comparison to baseline lactate alone. In addition, after adjusting for diabetes in a multivariate regression analysis, glucose was no independent predictor for in-hospital mortality.

We acknowledge several limitations in our study. Firstly, our reliance on electronic medical records may have resulted in incomplete or inaccurately recorded data, potentially affecting the validity of our findings. Secondly, the observational design limits our ability to establish causal relationships, and unmeasured confounding factors may influence the observed associations. Thirdly, the generalizability of our results is constrained by the single-center setting, which may not reflect broader patient populations or different healthcare environments. Finally, while our patients were classified within SCAI stages B-E, individual SCAI stages were not available. This limitation impacts the granularity of our prognostication.

## Conclusion

In this cohort of critically-ill patients with and without known diabetes, lactate at admission was superior for mortality prediction in comparison to glucose. Glucose, despite being implicated in energy metabolism, was not independently predictive for mortality.

## Supporting information

S1 Table Patient characteristics, dissected for AMI and non-AMI. (DOCX)

S2 Table Additional patient characteristics applying to patients who were admitted to intensive care unit (ICU). (DOCX)

S3 Table Restricted cubic splines model. (DOCX)

S1 Fig Decision curves for lactate and glucose cutoffs. (TIF)

## References

[1] Reynolds HR, Hochman JS. Cardiogenic Shock. Circulation. 2008;117(5):686–97. doi: 10.1161/CIRCULATIONAHA.106.613596 18250279

[2] Hunziker L, Radovanovic D, Jeger R, Pedrazzini G, Cuculi F, Urban P, et al. Twenty-Year Trends in the Incidence and Outcome of Cardiogenic Shock in AMIS Plus Registry. Circ Cardiovasc Interv. 2019;12(4):e007293. doi: 10.1161/CIRCINTERVENTIONS.118.007293 . 30943781

[3] Collet J-P, Thiele H, Barbato E, Barthélémy O, Bauersachs J, Bhatt DL, et al. 2020 ESC Guidelines for the management of acute coronary syndromes in patients presenting without persistent ST-segment elevation: The Task Force for the management of acute coronary syndromes in patients presenting without persistent ST-segment elevation of the European Society of Cardiology (ESC). European heart journal. 2020;42(14):1289–367. doi: 10.1093/eurheartj/ehaa575 32860058

[4] Hochman JS, Sleeper LA, White HD, Dzavik V, Wong SC, Menon V, et al. One-Year Survival Following Early Revascularization for Cardiogenic Shock. Jama. 2001;285(2):190–2. doi: 10.1001/jama.285.2.190 11176812

[5] Tarvasmäki T, Lassus J, Varpula M, Sionis A, Sund R, Køber L, et al. Current real-life use of vasopressors and inotropes in cardiogenic shock—adrenaline use is associated with excess organ injury and mortality. Critical Care. 2016;20(1):208. doi: 10.1186/s13054-016-1387-1 27374027 PMC4931696

[6] McDonagh TA, Metra M, Adamo M, Gardner RS, Baumbach A, Böhm M, et al. 2021 ESC Guidelines for the diagnosis and treatment of acute and chronic heart failure: Developed by the Task Force for the diagnosis and treatment of acute and chronic heart failure of the European Society of Cardiology (ESC) With the special contribution of the Heart Failure Association (HFA) of the ESC. European heart journal. 2021;42(36):3599–726. doi: 10.1093/eurheartj/ehab368 34447992

[7] Pöss J, Köster J, Fuernau G, Eitel I, Waha Sd, Ouarrak T, et al. Risk Stratification for Patients in Cardiogenic Shock After Acute Myocardial Infarction. Journal of the American College of Cardiology. 2017;69(15):1913–20. doi: 10.1016/j.jacc.2017.02.027 28408020

[8] Lazzeri C, Valente S, Chiostri M, Gensini GF. Clinical significance of lactate in acute cardiac patients. World J Cardiol. 2015;7(8):483–9. doi: 10.4330/wjc.v7.i8.483 ; PubMed Central PMCID: PMC4549782. 26322188 PMC4549782

[9] Fuernau G, Desch S, de Waha-Thiele S, Eitel I, Neumann F-J, Hennersdorf M, et al. Arterial Lactate in Cardiogenic Shock. JACC: Cardiovascular Interventions. 2020;13(19):2208–16. doi: 10.1016/j.jcin.2020.06.037 33032708

[10] Beer BN, Jentzer JC, Weimann J, Dabboura S, Yan I, Sundermeyer J, et al. Early risk stratification in patients with cardiogenic shock irrespective of the underlying cause – the Cardiogenic Shock Score. European Journal of Heart Failure. 2022;24(4):657–67. 10.1002/ejhf.2449. 35119176

[11] Melkonian EA, Schury MP. Biochemistry, anaerobic glycolysis. StatPearls Publishing, Treasure Island (FL). 2019. 31536301

[12] Hers H, Hue L. Gluconeogenesis and related aspects of glycolysis. Annual review of biochemistry. 1983;52(1):617–53. doi: 10.1146/annurev.bi.52.070183.003153 6311081

[13] Revelly JP, Tappy L, Martinez A, Bollmann M, Cayeux MC, Berger MM, et al. Lactate and glucose metabolism in severe sepsis and cardiogenic shock. Crit Care Med. 2005;33(10):2235–40. doi: 10.1097/01.ccm.0000181525.99295.8f . 16215376

[14] Levy B. Lactate and shock state: the metabolic view. Current opinion in critical care. 2006;12(4):315–21. doi: 10.1097/01.ccx.0000235208.77450.15 16810041

[15] Marbach JA, Di Santo P, Kapur NK, Thayer KL, Simard T, Jung RG, et al. Lactate Clearance as a Surrogate for Mortality in Cardiogenic Shock: Insights From the DOREMI Trial. Journal of the American Heart Association. 2022;11(6):e023322. doi: 10.1161/JAHA.121.023322 35261289 PMC9075306

[16] Rigamonti F, Montecucco F, Boroli F, Rey F, Gencer B, Cikirikcioglu M, et al. The peak of blood lactate during the first 24h predicts mortality in acute coronary syndrome patients under extracorporeal membrane oxygenation. International Journal of Cardiology. 2016;221:741–5. doi: 10.1016/j.ijcard.2016.07.065 27428314

[17] Park IH, Yang JH, Jang WJ, Chun WJ, Oh JH, Park YH, et al. Clinical significance of lactate clearance in patients with cardiogenic shock: results from the RESCUE registry. Journal of Intensive Care. 2021;9(1):63. doi: 10.1186/s40560-021-00571-7 34663479 PMC8522140

[18] Wang J, Ji M. The 6-h lactate clearance rate in predicting 30-day mortality in cardiogenic shock. Journal of Intensive Medicine. 2024. 10.1016/j.jointm.2024.01.003. PMC11258499 39035609

[19] Levy B, Girerd N, Baudry G, Duarte K, Cuau S, Bakker J, et al. Serial daily lactate levels association with 30-day outcome in cardiogenic shock patients treated with VA-ECMO: a post-hoc analysis of the HYPO-ECMO study. Annals of Intensive Care. 2024;14(1):43. doi: 10.1186/s13613-024-01266-6 38536534 PMC10973308

[20] Slottosch I, Liakopoulos O, Kuhn E, Scherner M, Deppe A-C, Sabashnikov A, et al. Lactate and lactate clearance as valuable tool to evaluate ECMO therapy in cardiogenic shock. Journal of Critical Care. 2017;42:35–41. doi: 10.1016/j.jcrc.2017.06.022 28672145

[21] Kliegel A, Losert H, Sterz F, Holzer M, Zeiner A, Havel C, et al. Serial Lactate Determinations for Prediction of Outcome After Cardiac Arrest. Medicine. 2004;83(5):274–9. doi: 10.1097/01.md.0000141098.46118.4c -200409000-00002. 15342971

[22] Düring J, Dankiewicz J, Cronberg T, Hassager C, Hovdenes J, Kjaergaard J, et al. Lactate, lactate clearance and outcome after cardiac arrest: A post-hoc analysis of the TTM-Trial. Acta Anaesthesiologica Scandinavica. 2018;62(10):1436–42. doi: 10.1111/aas.13172 29926901

[23] Dusik M, Rob D, Smalcova J, Havranek S, Karasek J, Smid O, et al. Serum lactate in refractory out-of-hospital cardiac arrest: Post-hoc analysis of the Prague OHCA study. Resuscitation. 2023;192:109935. doi: 10.1016/j.resuscitation.2023.109935 37574002

[24] Thoegersen M, Josiassen J, Helgestad OK, Berg Ravn H, Schmidt H, Holmvang L, et al. The association of diabetes and admission blood glucose with 30-day mortality in patients with acute myocardial infarction complicated by cardiogenic shock. European Heart Journal Acute Cardiovascular Care. 2020;9(6):626–35. doi: 10.1177/2048872620925265 32450719

[25] Yuan Y, Tao J, Shen X, Cheng H, Dong X, Muyesai N, et al. Elevated random glucose levels at admission are associated with all-cause mortality and cardiogenic shock during hospitalisation in patients with acute myocardial infarction and without diabetes: A retrospective cohort study. Diabetes/Metabolism Research and Reviews. 2023;39(4):e3617. doi: 10.1002/dmrr.3617 36729039

[26] Yang JH, Song PS, Song YB, Hahn J-Y, Choi S-H, Choi J-H, et al. Prognostic value of admission blood glucose level in patients with and without diabetes mellitus who sustain ST segment elevation myocardial infarction complicated by cardiogenic shock. Critical Care. 2013;17(5):R218. doi: 10.1186/cc13035 24090250 PMC4056011

[27] Kosiborod M, Rathore SS, Inzucchi SE, Masoudi FA, Wang Y, Havranek EP, et al. Admission Glucose and Mortality in Elderly Patients Hospitalized With Acute Myocardial Infarction. Circulation. 2005;111(23):3078–86. doi: 10.1161/CIRCULATIONAHA.104.517839 15939812

[28] Tada K, Nagao K, Tanjoh K, Hayashi N. Prognostic Value of Blood Glucose in Patients With Cardiogenic Shock. Circulation Journal. 2006;70(8):1064–9. doi: 10.1253/circj.70.1064 16864943

[29] Choi SH, Yoon G-S, Lee M-J, Park S-D, Ko Y-G, Ahn C-M, et al. Prognostic Impact of Plasma Glucose on Patients With Cardiogenic Shock With or Without Diabetes Mellitus from the SMART RESCUE Trial. The American Journal of Cardiology. 2022;175:145–51. doi: 10.1016/j.amjcard.2022.04.008 35550823

[30] Kataja A, Tarvasmäki T, Lassus J, Cardoso J, Mebazaa A, Køber L, et al. The association of admission blood glucose level with the clinical picture and prognosis in cardiogenic shock–Results from the CardShock Study. International Journal of Cardiology. 2017;226:48–52. doi: 10.1016/j.ijcard.2016.10.033 27788389

[31] Kattel S, Kasai T, Matsumoto H, Yatsu S, Murata A, Kato T, et al. Association between elevated blood glucose level on admission and long-term mortality in patients with acute decompensated heart failure. Journal of Cardiology. 2017;69(4):619–24. doi: 10.1016/j.jjcc.2016.05.013 27554050

[32] Abdin A, Pöss J, Fuernau G, Ouarrak T, Desch S, Eitel I, et al. Revision: prognostic impact of baseline glucose levels in acute myocardial infarction complicated by cardiogenic shock—a substudy of the IABP-SHOCK II-trial. Clinical Research in Cardiology. 2018;107(6):517–23. doi: 10.1007/s00392-018-1213-7 29423774

[33] Deane AM, Horowitz M. Dysglycaemia in the critically ill–significance and management. Diabetes, Obesity and Metabolism. 2013;15(9):792–801. 10.1111/dom.12078. 23368662

[34] Levraut J, Ciebiera JP, Chave S, Rabary O, Jambou P, Carles M, et al. Mild hyperlactatemia in stable septic patients is due to impaired lactate clearance rather than overproduction. Am J Respir Crit Care Med. 1998;157(4 Pt 1):1021–6. doi: 10.1164/ajrccm.157.4.9705037 . 9563714

[35] Marik PE, Bellomo R. Stress hyperglycemia: an essential survival response! Critical Care. 2013;17(2):305. doi: 10.1186/cc12514 23470218 PMC3672537

[36] Ishihara M, Inoue I, Kawagoe T, Shimatani Y, Kurisu S, Nishioka K, et al. Impact of acute hyperglycemia on left ventricular function after reperfusion therapy in patients with a first anterior wall acute myocardial infarction. American Heart Journal. 2003;146(4):674–8. doi: 10.1016/S0002-8703(03)00167-4 14564322

[37] Eitel I, Hintze S, Waha Sd, Fuernau G, Lurz P, Desch S, et al. Prognostic Impact of Hyperglycemia in Nondiabetic and Diabetic Patients With ST-Elevation Myocardial Infarction. Circulation: Cardiovascular Imaging. 2012;5(6):708–18. doi: 10.1161/CIRCIMAGING.112.974998 23051889

[38] Luo C, Chen F, Liu L, Ge Z, Feng C, Chen Y. Impact of diabetes on outcomes of cardiogenic shock: A systematic review and meta-analysis. Diab Vasc Dis Res. 2022;19(5):14791641221132242. doi: 10.1177/14791641221132242 ; PubMed Central PMCID: PMC9580099. 36250870 PMC9580099

[39] Zeller M, Cottin Y, Brindisi M-C, Dentan G, Laurent Y, Janin-Manificat L, et al. Impaired fasting glucose and cardiogenic shock in patients with acute myocardial infarction. European heart journal. 2004;25(4):308–12. doi: 10.1016/j.ehj.2003.12.014 14984919

[40] Shindler Daniel M, Palmeri Sebastian T, Antonelli Tracy A, Sleeper Lynn A, Boland J, Cocke Thomas P, et al. Diabetes mellitus in cardiogenic shock complicating acute myocardial infarction: a report from the SHOCK Trial Registry. Journal of the American College of Cardiology. 2000;36(3_Supplement_1):1097–103. doi: 10.1016/S0735-1097(00)00877-9 10985711

