# Supplementary material for: Blood glucose and lactate levels as early predictive markers in patients presenting with cardiogenic shock: A retrospective cohort study
Source: PLoS One. 2024 Jul 25;19(7):e0306107. doi: 10.1371/journal.pone.0306107 (PMC11271948; doi:10.1371/journal.pone.0306107)
Supplement: S1 Table — (DOCX) [file pone.0306107.s001.docx]

**S1 table 1: Patient characteristics, dissected for AMI and non-AMI**

|  | **AMI (n: 143)** | **Non-AMI (n: 169)** | **p-value^a^** |
| --- | --- | --- | --- |
| Sex (male), n (%) | 115 (80.4) | 124 (73.4) | 0.143 |
| Age | 71 (61-78) | 70 (59-80) | 0.876 |
| Discharged alive, n (%) | 58 (40.6) | 43 (25.4) | **0.004** |
| Left ventricular ejection fraction [%] | 38 (29-45) | 40 (20-55) | 0.532 |
| Cardiac Arrest at presentation, n (%) | 86 (60.1) | 121 (72) | **0.027** |
| Bystander resuscitation, n (%) | 39 (37.5) | 39 (27.7) | 0.102 |
| On mechanical ventilation, n (%) | 99 (72.8) | 128 (80.5) | 0.117 |
| Mechanical circulatory support |  |  |  |
| Use of any MCS, n (%) | 49 (34.4) | 18 (10.7) | **< 0.001** |
| - Impella, n (%) | 37 (25.9) | 10 (5.9) |  |
| - IABP, n (%) | 1 (0.7) | 0 |  |
| - VA-ECMO, n (%) | 9 (6.3) | 5 (3) |  |
| - ECMELLA, n (%) | 2 (1.4) | 3 (1.8) |  |
| Medical history |  |  |  |
| Ischemic heart disease, n (%) | 35 (24.5) | 51 (30.2) | 0.261 |
| Heart Failure, n (%) | 19 (15.4) | 31 (26.3) | **0.038** |
| Diabetes, n (%) | 42 (33.9) | 39 (32.8) | 0.856 |
| Arterial hypertension, n (%) | 78 (62.4) | 80 (64) | 0.793 |
| Ever-Smoker, n (%) | 47 (38.2) | 36 (30.8) | 0.226 |
| Laboratory findings |  |  |  |
| GFR [ml/min] | 70 (40-70) | 54 (23-70) | 0.16 |
| ALT [U/l] | 78 (36-187) | 77 (39-204) | 0.492 |
| White blood cell count [G/l] | 13.3 (10.2-19.6) | 12.6 (9.1-16.6) | 0.097 |
| Haemoglobin [g/dl] | 12.4 (10.8-14) | 11.9 (10.1-13.9) | 0.304 |
| pH | 7.29 (7.11-7.4) | 7.14 (6.87-7.35) | **< 0.001** |
| Glucose [mg/dl] | 227 (153-302) | 235 (154-366) | 0.301 |
| Lactate [mmol/l] | 5.4 (2.4-10.4) | 8.92 (3.76-12.23) | **< 0.001** |
| Vital signs after ICU admission |  |  |  |
| Mean arterial pressure [mmHg] | 76 (65-89) | 78 (66-90) | 0.674 |
| Heart rate [BPM] | 82 (68-100) | 84 (84-113) | 0.111 |

AMI acute myocardial infarction

^a^Bonferroni correction was used to correct for multiple testing,
