# Supplementary material for: Blood glucose and lactate levels as early predictive markers in patients presenting with cardiogenic shock: A retrospective cohort study
Source: PLoS One. 2024 Jul 25;19(7):e0306107. doi: 10.1371/journal.pone.0306107 (PMC11271948; doi:10.1371/journal.pone.0306107)
Supplement: S2 Table — (DOCX) [file pone.0306107.s002.docx]

**S2 table 2: Additional patient characteristics applying to patients who were admitted to intensive care unit (ICU)**

|  | **Survivors** | **Non-survivors** | **p-value^a^** |
| --- | --- | --- | --- |
| Admitted to ICU, n (%) | 101 (100) | 126 (59.7) | **< 0.001** |
| Vasopressors or inotropes use during the first 3h on ICU, n (%) | 76 (75.2) | 105 (80.8) | 0.312 |
| - number of vasopressors or inotropes used: |  |  |  |
| - 1 | 23 (22.8) | 25 (19.8) |  |
| - 2 | 47 (46.5) | 49 (38.9) |  |
| - 3 | 6 (5.9) | 23 (18.3) |  |
| - 4 | 0 | 2 (1.6) |  |
| - 5 | 0 | 2 (1.6 |  |
| Dialysis during the first 24h on ICU, n (%) | 84 (84) | 68 (54.8) | **< 0.001** |
| Targeted temperature management, n (%) | 35 (36.8) | 57 (50.4) | **0.049** |
| Cerebral hypoxia, n (%) | 1 (1) | 20 (16.1) | **< 0.001** |
| Stroke, n (%) | 1 (1) | 5 (4.1) | 0.159 |
| Intracerebral bleeding, n (%) | 1 (1) | 4 (3.2) | 0.262 |

^a^Bonferroni correction was used to correct for multiple testing,

ICU intensive care unit
