# Supplementary material for: Blood glucose and lactate levels as early predictive markers in patients presenting with cardiogenic shock: A retrospective cohort study
Source: PLoS One. 2024 Jul 25;19(7):e0306107. doi: 10.1371/journal.pone.0306107 (PMC11271948; doi:10.1371/journal.pone.0306107)
Supplement: S3 Table — (DOCX) [file pone.0306107.s003.docx]

**S4 Table 3. Restricted cubic splines model.**

| **Predictor** | **Coefficient** | **95% CI Lower** | **95% CI Upper** | **Hazard Ratio** | **p-value** | **Non-linear p** |
| --- | --- | --- | --- | --- | --- | --- |
| Baseline lactate | 0.923 | 0.448 | 1.397 | 2.516 | **< 0.001** | 0.579 |
| Baseline glucose | -0.150 | -0.580 | 0.280 | 0.861 | 0.445 | 0.443 |
| Age | 0.414 | 0.182 | 0.647 | 1.514 | **< 0.001** |  |
| Male Sex | 0.312 | -0.026 | 0.651 | 1.367 | 0.071 |  |
| Myocardial infarction | -0.019 | -0.317 | 0.280 | 0.981 | 0.902 |  |
| Diabetes mellitus | 0.404 | 0.084 | 0.724 | 1.498 | **0.013** |  |
| Cardiac arrest at presentation | -0.371 | -0.727 | -0.015 | 0.690 | **0.041** |  |
| Baseline pH | -0.486 | -0.833 | -0.140 | 0.615 | **0.006** |  |
| Baseline hemoglobin | -0.290 | -0.519 | -0.061 | 0.748 | **0.013** |  |
| Heart rate | 0.203 | -0.047 | 0.454 | 1.226 | 0.111 |  |
| Mean arterial pressure | 0.004 | -0.186 | 0.195 | 1.004 | 0.965 |  |

CI conficende interval
