# Supplementary figures and images for: Blood glucose and lactate levels as early predictive markers in patients presenting with cardiogenic shock: A retrospective cohort study
Source: PLoS One. 2024 Jul 25;19(7):e0306107. doi: 10.1371/journal.pone.0306107 (PMC11271948; doi:10.1371/journal.pone.0306107)

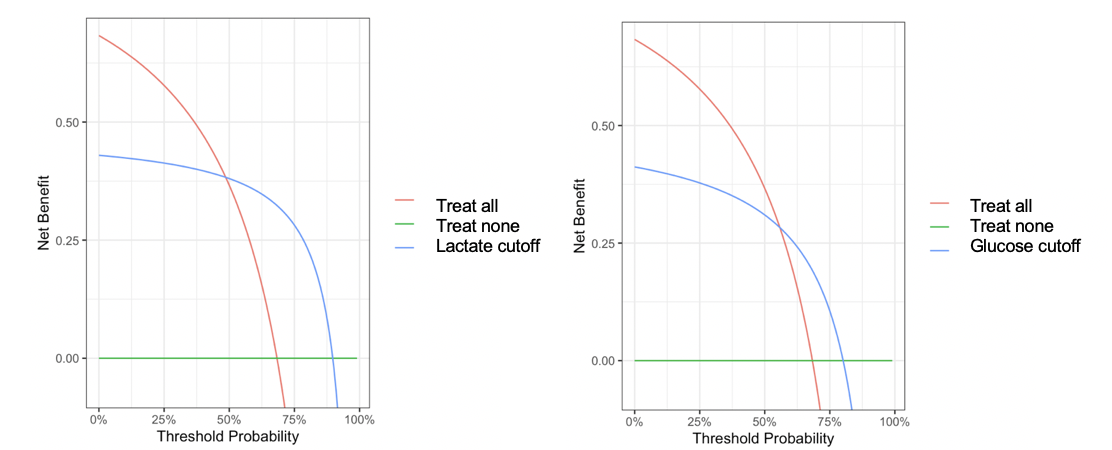

Supplement: S1 Fig — (TIF) [file pone.0306107.s004.tif]
